# Enhancement of the Modulation Response of Quantum-Dot-Based Down-Converted Light through Surface Plasmon Coupling

**DOI:** 10.3390/molecules27061957

**Published:** 2022-03-17

**Authors:** Shaobo Yang, Po-Yu Chen, Chia-Chun Ni, Jun-Chen Chen, Zong-Han Li, Yang Kuo, Chih-Chung Yang, Ta-Cheng Hsu, Chi-Ling Lee

**Affiliations:** 1 Institute of Photonics and Optoelectronics, Department of Electrical Engineering, National Taiwan University, No. 1, Section 4, Roosevelt Road, Taipei 10617, Taiwan; f08941106@ntu.edu.tw (S.Y.); r06941113@ntu.edu.tw (P.-Y.C.); r06941105@ntu.edu.tw (C.-C.N.); r08941074@ntu.edu.tw (J.-C.C.); r08941073@ntu.edu.tw (Z.-H.L.); 2 Department of Energy and Refrigerating Air-Conditioning Engineering, Tungnan University, 152 Beishen Road, Section 3, New Taipei City 22202, Taiwan; ykuo@mail.tnu.edu.tw; 3 Unikorn Semiconductor Corp., No. 5, Li-hsin 5th Rd., Hsinchu Science Park, Hsinchu 30078, Taiwan; tc_hsu@unikornsemi.com; 4 Epistar Corp., No. 5, Li-hsin 5th Rd., Hsinchu Science Park, Hsinchu 30078, Taiwan; kirin_lee@epistar.com

**Keywords:** surface plasmon, modulation bandwidth, photon down-conversion, colloidal quantum dot, light-emitting diode, Förster resonance energy transfer

## Abstract

In this paper, we first elaborate on the effects of surface plasmon (SP) coupling on the modulation responses of the emission of a light-emitting diode (LED) and its down-converted lights through colloidal quantum dots (QDs). The results of our past efforts for this subject are briefly discussed. The discussions lay the foundation for the presentation of the new experimental data of such down-converted lights in this paper. In particular, the enhancement of the modulation bandwidth (MB) of a QD-based converted light through SP coupling is demonstrated. By linking green-emitting QDs (GQDs) and/or red-emitting QDs (RQDs) with synthesized Ag nano-plates via surface modifications and placing them on a blue-emitting LED, the MBs of the converted green and red emissions are significantly increased through the induced SP coupling of the Ag nano-plates. When both GQD and RQD exist and are closely spaced in a sample, the energy transfer processes of emission-reabsorption and Förster resonance energy transfer from GQD into RQD occur, leading to the increase (decrease) in the MB of green (red) light. With SP coupling, the MB of a mixed light is significantly enhanced.

## 1. Introduction

The modulation response of a light-emitting device is an important issue for study when the device is used for communication purposes. A faster modulation response or a larger modulation bandwidth (MB) of such a device can lead to a higher communication capability. Recently, visible communication has found broad applications in the areas of indoor links and autonomous cars [1,2,3,4]. Because of their low cost, light-emitting diodes (LEDs) have been considered as the light sources for visible communications. In particular, a visible communication system can be combined with a lighting facility of white light. Therefore, the maximization of the MB of a single- or mixed-color device is an important task in LED design and fabrication [5,6,7,8,9,10,11,12,13,14]. In a mixed-color or white-light LED, photon down-conversion is involved in producing multiple colors in the same device [15,16,17]. The MB of a mixed-color LED depends on the intensity fractions of different contributing colors. Because the MB of the fundamental color (typically blue light) is usually larger, when compared with that of a converted light, a higher intensity fraction from the fundamental color in a multi-color light source can lead to a larger overall MB [7]. However, such a design sets a limit to the choice of color rendering index or white-light quality. Therefore, for implementing a large MB in a multi-color LED, efforts for increasing the MBs of individual light-color components are needed. In particular, the improvement of the modulation response of a converted light requires more attention because it is delayed in the conventional color conversion process.

The modulation response of a light emitter is controlled by the rise and fall times of emitted light under a modulated electrical excitation. Typically, the rise time is significantly shorter than 1 ns and does not limit the required MB for the current applications. The research focus usually aims at the reduction of the fall time, i.e., the time constant to switch off the emission after the electrical excitation is turned off. For this purpose, surface plasmon (SP) coupling is a simple and effective approach [18,19,20,21,22,23,24,25,26,27]. SP resonance can be excited at the interface between two materials when the real part of the dielectric constant of the material on one side is negative while that on the other side is positive [28]. Such a phenomenon can be observed typically at the interface between a metal nanostructure and a surrounding dielectric medium. In certain metals, such as Al, Ag, and Au, the real parts of the dielectric constants are negative in the visible range. Therefore, we can excite SP resonance at the interface between a nanostructure of such a metal and GaN, which is used for fabricating visible LEDs and can be regarded as a dielectric medium in the visible range (below its bandgap). In other words, a metal nanostructure can be fabricated on or inside a GaN-based LED device for producing SP coupling with its InGaN/GaN quantum wells (QWs) and/or other added light emitters. Typically, SP coupling can be classified into the categories of strong and weak couplings, depending on whether a significant Rabi splitting is produced. If a significant Rabi splitting is observed in an SP coupling process, it falls into the category of strong coupling [29]; otherwise, it is a weak coupling process. In this paper, only weak SP coupling is concerned for the targeted application.

Although SP coupling in an LED can produce many other advantages for its performance, in this paper, we will focus on the SP coupling effects on the enhancement of LED modulation response. In particular, we will investigate the improvement of the MB of the color-converted light through overlaid colloidal quantum dots (QDs). In this paper, we first discuss the fundamentals of SP coupling and the enhancements of the modulation responses of LED emission and color-converted light through SP coupling in Section 2. The mechanisms of such enhancements are explained. Then, in Section 3, we present the experimental results of the MB enhancements of the green and red lights obtained from the down-conversion based on a blue-emitting LED overlaid with colloidal QDs. It is shown that with the SP coupling through linked synthesized Ag nanoparticles (NPs), the MBs of the green and red lights are significantly increased. In this regard, the Förster resonance energy transfer (FRET) from the green into red light plays an important role [30]. This part of the presentation includes the LED device structures and fabrication procedures in Section 3.1, the modulation behaviors of the emitted lights in Section 3.2, and the discussions about the results in Section 3.3. The use of QD-linked synthesized metal NPs on an LED to induce SP coupling effects represents a new and effective technique for enhancing the MB of a down-converted light. Finally, conclusions of the whole paper are drawn in Section 4.

## 2. Surface Plasmon Coupling for Enhancing Modulation Response

### 2.1. Fundamentals of Surface Plasmon Coupling

**Figure 1:**
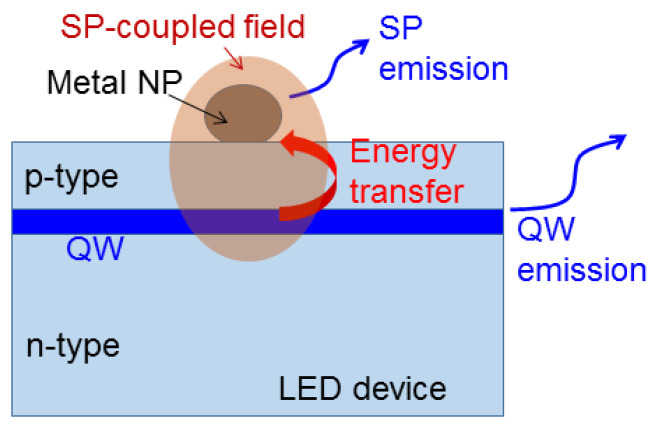
Schematic illustration of an SP coupling process between the SP resonance of a surface metal NP and a QW in an LED device showing the energy transfer from the QW into the SP resonance.

SP coupling can be understood as one kind of Purcell effect, in which the SP resonance field of a metal nanostructure can enhance the emission efficiency of a light emitter nearby [31]. Although the dissipation of the metal nanostructure can produce a certain energy loss, the overall emission efficiency of the metal-emitter system can still be increased. With SP coupling, both radiative and non-radiative processes of this system become stronger, leading to a higher decay rate of photoluminescence (PL) or carrier concentration in a semiconductor light emitter, such as an InGaN/GaN QW or a CdZnSeS/ZnS colloidal QD. An SP coupling process can also be interpreted as the transfer of the energy of a nearby light emitter into the SP resonance of a metal nanostructure. Part of the SP resonance energy radiates and the rest is dissipated by metal. Such an energy transfer occurs with a non-radiative recombination process of carriers in a semiconductor light emitter. The radiation of SP resonance coherently adds to that of the light emitter to enhance the overall emission efficiency. With this energy transfer, the decay rate of carrier concentration in this light emitter becomes higher. By considering an InGaN/GaN QW in an LED device as the light emitter for coupling with the SP resonance of a surface metal NP, in Figure 1, we schematically illustrate the energy flow paths in such an SP coupling process. In this process, both the coupled portion of the QW and the metal NP are covered by the SP-coupled electromagnetic field distribution, through which energy in the QW can be transferred into the SP resonance mode. Part of the transferred energy is used for SP emission. Part of the energy that remained in the QW is used for QW emission.

With the excitation condition fixed, in an SP coupling process, the energy of the emission enhancement originates from that of the intrinsic defect-induced non-radiative recombination. In other words, the intrinsic non-radiative recombination energy in a QW or QD is converted into photon emission through SP coupling [32]. Therefore, SP coupling can enhance the internal quantum efficiency (IQE) of a QW or QD [33,34,35,36,37,38,39,40]. Based on the assumption that the non-radiative recombination is negligibly weak at a low temperature (4–10 K), IQE can be defined as the ratio of the integrated PL intensity at room temperature (300 K) over that at the low temperature. For a light emitter with a higher intrinsic IQE, the effect of SP coupling for IQE enhancement becomes weaker. In other words, SP coupling for enhancing emission efficiency is particularly useful for those light emitters of low intrinsic emission efficiencies.

As mentioned earlier, SP coupling can lead to a higher carrier decay rate in a QW or QD. Therefore, the response to the excitation switching-off of such a light emitter is expected to be faster. In other words, the MB of such a light-emitting device can be increased through SP coupling [41,42,43]. Meanwhile, with the higher decay rate of carrier concentration in a QW under the SP coupling condition, the carrier concentration in the QW becomes lower. The lower carrier concentration can help in reducing the effects of carrier overflow and Auger recombination. Carrier overflow means the reduction of the carrier capture capability of a QW in an LED when the carrier concentration in the QW is high. The Auger recombination rate is proportional to the cube of carrier concentration and hence its effect is strong when the carrier concentration is high. Carrier overflow and Auger recombination are two major mechanisms for producing the efficiency droop behavior of an LED, in which the LED efficiency decreases with increasing injected current after it passes an efficiency maximum at a low injected current level. This behavior makes the fabrication of a high-power LED difficult. With SP coupling in an LED, the carrier concentration in its QWs can be significantly decreased, and hence its efficiency droop behavior can be suppressed for high-power LED fabrication [44,45,46].

SP coupling can be implemented with various metal nanostructures [47,48,49]. However, the simplest and most inexpensive metal nanostructure for realizing a strong SP coupling effect is metal NP. Metal NPs can be fabricated on a device surface through thin-layer metal deposition and then thermal annealing. Metal NPs immersed in a solution can also be fabricated through chemical synthesis [50,51]. The surface charges of chemically synthesized metal NPs can be modified such that they can link with colloidal QDs through electrostatic attraction. The synthesized metal NPs can also have the flexibility of inserting into nanoscale cavities in a device for producing stronger SP coupling. Among different metals for producing SP coupling, Ag is a good choice for SP coupling in the visible range due to its favored plasma frequency and low dissipation. Deposited Ag NPs on the top surface have been used for demonstrating the SP coupling effects to increase the IQE and MB, and to suppress the efficiency droop effect of an LED [41,42,43,44,45,46]. In this regard, a key issue is the distance between the surface Ag NPs and the QWs of the LED. For effective SP coupling, typically this distance needs to be smaller than ~100 nm. Generally, a shorter distance leads to a stronger SP coupling effect. This distance condition sets a limit to the thickness of the p-type layer in an LED, including the p-AlGaN electron blocking layer and the p-GaN current spreading layer. However, a smaller p-GaN layer thickness results in an increase in device resistance. A compromise between the SP coupling effect and the device’s electrical property needs to be made. It has been demonstrated that by reducing the distance between the surface Ag NPs and the top QW down to ~66 nm, which includes ~25-nm top quantum barrier layer, ~15 nm p-AlGaN layer, ~8 nm p-GaN layer, ~8 nm p^+^-GaN layer, and ~10 nm GaZnO transparent conducting layer, the degradation of LED electrical property is still acceptable [52]. In this situation, the LED efficiency increase due to SP coupling can overcompensate the performance degradation caused by the poorer electrical property [53,54]. Therefore, with an appropriate structure design, SP coupling can effectively enhance the emission efficiency of an LED. Although the SP coupling effect for enhancing the emission efficiency of an LED decreases with increasing intrinsic efficiency, it has been demonstrated that the IQE of a commercial-standard blue LED of ~80% in intrinsic IQE can be increased to ~90% under a carefully designed SP coupling condition [52]. For effective SP coupling, the SP resonance spectrum needs to overlap the QW emission wavelength. The SP resonance spectrum of a metal NP can be adjusted by controlling its shape and size. It can also be tuned through the change of its surrounding material. For instance, by inserting a thin dielectric layer of a lower refractive index between surface metal NPs and the LED top layer, the SP resonance spectrum can be blue-shifted [43,45,46,55]. On the other hand, by covering the surface metal NPs with a certain dielectric material, the SP resonance spectrum can be red-shifted.

### 2.2. Modulation Response Enhancement of a Light-Emitting Diode through SP Coupling

Besides the device circuit factor, i.e., the RC time constant, the modulation response of a semiconductor light-emitting device is controlled by three factors, including the decay rate of its carrier concentration, the injected rate of the carrier, and the thickness of its active layer. In a QW LED, these three factors are lumped into the time constant, *τ* as [56]

*τ* = (*qd*/*JB*)^1/2^. (1)

Here, *q* is the electron charge, *d* is the thickness of the active layer, *J* is the injected current density, and *B* is the carrier decay rate in the QWs. The MB of an LED is inversely proportional to the time constant *τ*. Therefore, to increase the MB, we can increase the injected current density, decrease the number of QW periods, or increase the carrier decay rate in the QWs of the LED. Since SP coupling can increase the carrier decay rate of a semiconductor light emitter, it is a useful approach for increasing the MB of a light emitter. As mentioned above, the RC time constant is another factor controlling the MB of a device. A smaller RC time constant (smaller capacitance, C, and/or smaller resistance, R) leads to a faster modulation response or a higher MB. In an LED, the device capacitance (resistance) decreases (increases) with decreasing p-type mesa size. However, the decreasing range of the capacitance is larger than the increasing range of the resistance. Therefore, the RC time constant decreases with decreasing mesa size. In other words, the RC time constant is smaller and the MB is larger in a micro-LED when compared with a regular-size LED. An MB increase induced by SP coupling has been demonstrated [18,19,20,21,22,23,24,25,26,27,41,42,43]. Generally, the increase factor of MB is about the same as that of the square root of PL decay rate, indicating that the key SP coupling to increasing the MB is the enhancement of carrier decay rate in the QWs of an LED. Similar to the SP coupling-induced IQE enhancement, the increase factor of the MB is smaller in an LED of a larger intrinsic MB. Based on a commercial-standard QW structure, an LED of 66 nm in the distance between the top surface and the top QW and of 20 nm in circular mesa diameter was fabricated to achieve the increase in its MB from ~650 MHz to ~722 MHz [52].

### 2.3. Modulation Response in Photon Down-Conversion

**Figure 2:**
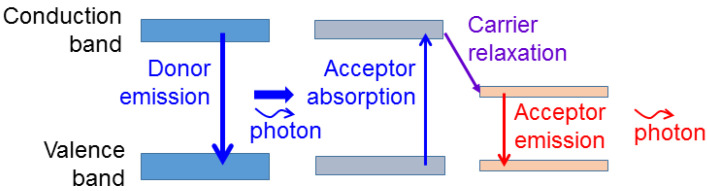
Schematic illustration of a conventional photon down-conversion process, including the four steps of donor emission, acceptor absorption, carrier relaxation, and acceptor emission.

The process of photon down-conversion involves four steps, including the emission of the energy donor, the absorption of donor emission by the energy acceptor, the relaxation of carriers from the absorption level into the emission level of the acceptor, and the emission of the acceptor. These four steps are schematically illustrated in Figure 2. Here, when both donor and acceptor are semiconductor light emitters, the upper and lower energy bands usually correspond to the conduction and valence bands, respectively. Such a four-step process can slow down the modulation response of converted light. Therefore, when a photon down-converted light is used to mix with the fundamental photon for white-light generation and the white light is modulated for a communication application, the MB is expected to be reduced. It has been demonstrated that SP coupling can be used for enhancing the photon down-conversion efficiency [57,58,59,60,61]. Basically, SP coupling not only can enhance the emission efficiency of a light emitter but also can increase the absorption efficiency of a light absorber. The application of a metal nanostructure to a solar cell device utilizes this function of SP coupling [62,63,64,65]. Therefore, SP coupling can be used for increasing the efficiencies of the three steps of light emission and absorption in a photon down-conversion process. In particular, the acceptor absorption cross-section can be significantly increased based on the near-field interactions of the donor and acceptor. When the acceptor emission efficiency is increased, the carrier relaxation rate in the acceptor can also be enhanced. Such near-field interactions include not only SP coupling but also FRET. FRET can be interpreted as the absorption of the near-field energy of the donor by the acceptor if the acceptor absorption spectrum covers the donor emission wavelength [30,66,67,68,69,70]. Since the near-field distribution decays fast with the distance from the donor, effective FRET can usually be observed only when the distance between the donor and acceptor is smaller than a few tens of nanometers. FRET is a useful channel for increasing the acceptor absorption cross-section. SP coupling can either enhance or reduce FRET efficiency [69,70,71,72,73,74,75]. However, the transferred power in a FRET process can usually be increased through SP coupling.

**Figure 3:**
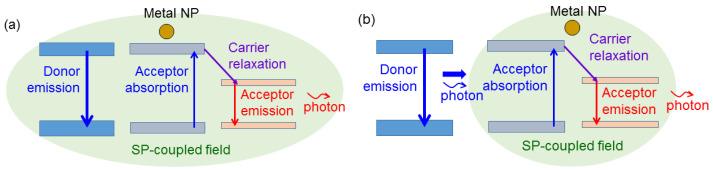
(**a**) Schematic illustration of a “three-body” SP-coupled down-conversion process when the SP resonance spectrum covers both emission wavelengths of the donor and acceptor while the donor, acceptor, and metal NP are close to each other. (**b**) Schematic illustration of an SP-coupled down-conversion process when the SP resonance spectrum covers both emission wavelengths of the donor and acceptor but the donor and acceptor are far apart.

If the SP resonance spectrum is broad enough to cover both emission spectra of the donor and acceptor, a “three-body coupling” process among the metal nanostructure of SP resonance, the donor, and acceptor can be induced to effectively transfer energy from the donor to acceptor [60]. As schematically illustrated in Figure 3a, in this coupling process, not only the emission efficiencies of both donor and acceptor can be increased, but also the acceptor absorption can be enhanced through the direct absorption of the near-field energy built by the SP coupling with the donor. In this figure, we use the greenish-shaded region to schematically illustrate the spatial and spectral coverages of the SP-coupled resonance electromagnetic field. In the case of using metal NPs for SP coupling, the broad homogeneous broadening of the SP resonance due to its high-loss nature plus the broad inhomogeneous broadening due to the distribution of the metal NPs of slightly different geometries can provide us with a broad SP resonance spectrum for simultaneously covering the emission wavelengths of the donor and acceptor, e.g., blue- and red-light emitters, respectively. For photon down-conversion in an LED device, i.e., converting the energy of the blue-emitting QWs into green or red light through colloidal QDs, efforts are needed to make the QWs close to the SP-resonance metal NPs and color-converting QDs even though it is not difficult to make the metal NPs close to the QDs. In the situation that the QWs are far away (>100 nm) from the metal NPs and QDs, the intensity of the far-field radiation of the QWs can still be enhanced through the SP resonance at the QW (donor) emission wavelength for increasing the absorbed power of the QDs (acceptor), as schematically illustrated in Figure 3b. Therefore, as long as the SP resonance spectrum is broad enough, SP coupling can effectively enhance the acceptor absorption and emission, and hence increase the overall efficiency of photon down-conversion. In the case of three-body coupling illustrated in Figure 3a, under the SP couplings with the donor and acceptor, their modulation responses are accelerated, leading to the overall increase in the MB of acceptor emission. In the case of the SP coupling illustrated in Figure 3b, the modulation response of the acceptor is accelerated to enhance the MB of the converted light. Meanwhile, the carrier relaxation rate will be increased due to the reduced upper-state population in the acceptor that can also help in speeding up the modulation response of the whole system.

**Figure 4:**
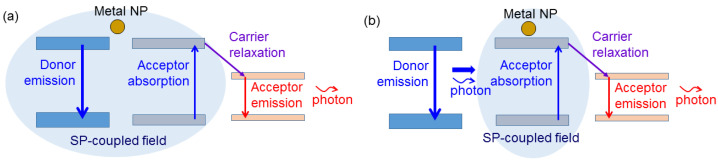
(**a**) Schematic illustration of an SP-coupled down-conversion process when the SP resonance spectrum covers only the emission wavelength of the donor, and the donor, acceptor, and metal NP are close to each other. (**b**) Schematic illustration of an SP-coupled down-conversion process when the SP resonance spectrum covers only the emission wavelength of the donor, and the donor and acceptor are far apart.

**Figure 5:**
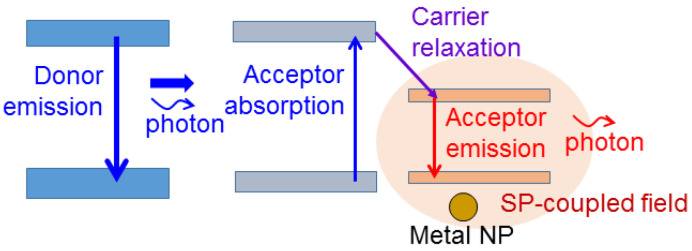
Schematic illustration of an SP-coupled down-conversion process when the SP resonance spectrum covers only the emission wavelength of the acceptor.

Under the condition that the SP resonance spectrum is narrow and covers only the donor emission wavelength, when the donor, acceptor, and metal NP are close to each other, as schematically illustrated in Figure 4a, the donor emission and acceptor absorption can be enhanced. Moreover, the modulation response of the donor emission is speeded up which can lead to the MB increase in acceptor emission. When the donor and acceptor are far apart, as schematically illustrated in Figure 4b, the acceptor absorption can be enhanced. In this situation, the SP coupling does not much help in enhancing the MB of acceptor emission. Under the condition that the SP resonance spectrum is narrow and covers only the acceptor emission wavelength, as schematically illustrated in Figure 5, only the acceptor emission can be enhanced. In this situation, the modulation response of the acceptor emission can be accelerated, leading to a higher MB of the converted light. Meanwhile, because of the faster consumption of the upper-state population in the acceptor, the absorption of donor emission and the relaxation of carriers from the absorption level into the emission level in the acceptor become more effective such that its modulation response can also become faster. Due to the more uniform geometries of chemically synthesized metal NPs, the SP resonance spectrum of such a metal NP sample is relatively narrower, when compared with a surface metal NP distribution fabricated through metal deposition and thermal annealing. Therefore, the SP coupling conditions illustrated in Figure 4 and Figure 5 can be observed when synthesized metal NPs are used for inducing SP coupling in a photon down-conversion device.

To make QDs close to metal NPs or different kinds of QDs, the surface coatings of certain molecules on QDs and metal NPs can change their surface charges such that they can attract each other for maintaining short distances [59,61,69]. Typically, a QD is capped with an amphiphilic polymer, such as poly(isobutylene-alt-maleic anhydride), and hence are negatively charged [76]. The surface of a QD can be modified by coating poly(sodium 4-styrenesulfonate) (PSS) and poly(allylamine hydrochloride) (PAH) molecules to become negatively and positively charged, respectively [77]. Chemically synthesized metal NPs are usually capped with polyethylene glycol (thiol-PEG-amine) (PEG) to avoid their aggregation and make the NPs positively charged [78]. PAH and PSS can also be applied to metal NPs for changing their surface charges. The linkage between different kinds of QDs, such as green- and red-emitting QDs, through their opposite surface charges, can make their mutual distances as small as several nanometers to guarantee effective FRET. A small mutual distance can also be achieved between linked metal NPs and QDs for producing a strong SP coupling effect [59,61,69]. However, a relatively larger distance (larger than ~15 nm) between a metal NP and a QD is preferred to avoid the process of direct electron transfer [69,79]. In this process, electrons in a light emitter (QD) can transport into a nearby metal structure through a certain tunneling channel such that the emission of the light emitter is reduced. The controls of the distances between metal NPs and QDs, between QDs and QDs, and between QWs and QDs or metal NPs are important issues for effective photon down-conversion. If a nanoscale cavity close to the QWs of a device can be fabricated for confining metal NPs and QDs, the mutual distances between the QWs, QDs, and metal NPs can be better controlled for near-field interactions [20,80]. In such a nanoscale cavity, the emission of a light emitter can be enhanced through a cavity effect. This nanoscale-cavity effect can also enhance the FRET efficiency [80].

## 3. Experimental Demonstrations

In this section, we report the experimental results of the MB enhancements of QD-converted green and red lights from a blue-emitting LED through the SP coupling of synthesized Ag nano-plates. With both green-emitting QD (GQD) and red-emitting QD (RQD) existent on an LED, the effects of the FRET from GQD into RQD on the MBs of green and red lights are also demonstrated.

### 3.1. Device Structures and Fabrication Procedures

**Figure 6:**
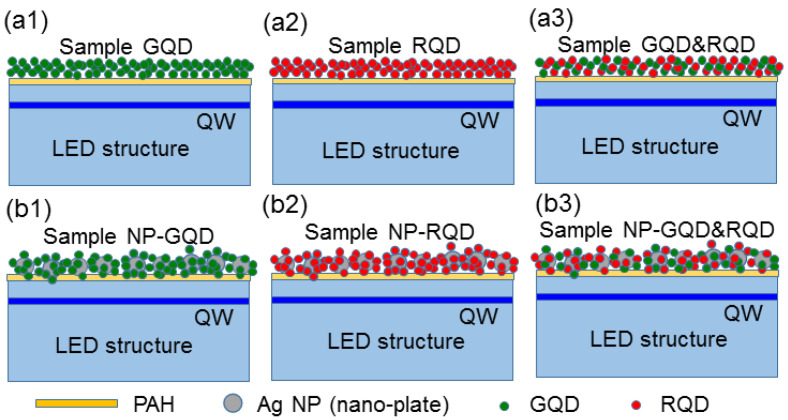
(**a1**)–(**a3**) and (**b1**)–(**b3**) Schematic illustrations of the surface structures of samples GQD, RQD, GQD&RQD, NP-GQD, NP-RQD, and NP-GQD&RQD, respectively.

The LED epitaxial structure, with the emission wavelength at ~450 nm, is provided by the Epistar Corporation, Hsinchu, Taiwan. The fabrication of the LED devices follows the standard process procedure with the square mesa size at 300 µm × 300 µm. The p-contact is located at the center of the mesa with metals Ni/Au of 20/100 nm in thickness. The n-contact is located around the p-type mesa with metals Ti/Au of 20/100 nm in thickness. The used GQDs and RQDs are purchased from Taiwan Nanocrystals Inc. Hsinchu, Taiwan. The CdZnSeS/ZnS GQD and RQD are capped with poly(isobutylene-alt-maleic anhydride) and are negatively charged with zeta potentials at −28.3 and −25.6 mV, respectively, which are measured with a zeta potential analyzer (Malvern Panalytical, Malvern, UK, Zetasizer Nano series Nano—Z) [76]. The emission peak wavelength of GQD (RQD) is ~520 (~625) nm. Figure 6(a1–a3) schematically shows the structures of samples GQD, RQD, and GQD&RQD, in which the fabricated LEDs are overlaid with GQD only, RQD only, and the mixture of GQD and RQD, respectively. Before GQD and/or RQD drop-casting, the LED is over-coated with PAH molecules for making its surface positively charged such that it can attract negatively charged QDs [77]. In fabricating sample GQD (RQD), we drop-cast a GQD (RQD) solution of 200 (50) µL in volume and 1 g/L in concentration with a culture insert of 1 × 1 cm in size and wait until it dries up naturally. For sample GQD&RQD, we drop-cast the mixture of 50 µL RQD solution and 200 µL GQD solution onto an LED.

**Figure 7:**
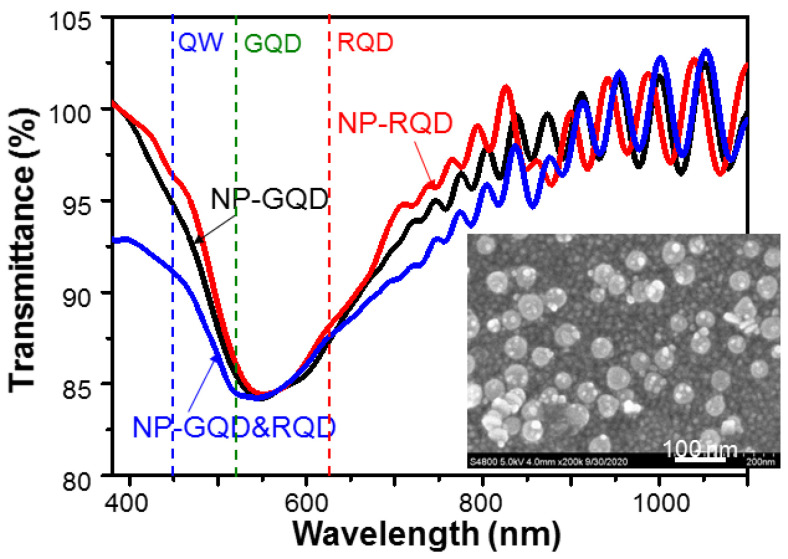
Transmission spectra of the surface structures in samples NP-GQD, NP-RQD, and NP-GQD&RQD. The vertical dashed lines show the emission wavelengths of the QWs of the LED, GQDs, and RQDs. The insert shows the SEM image of sample NP-GQD&RQD.

**Figure 8:**
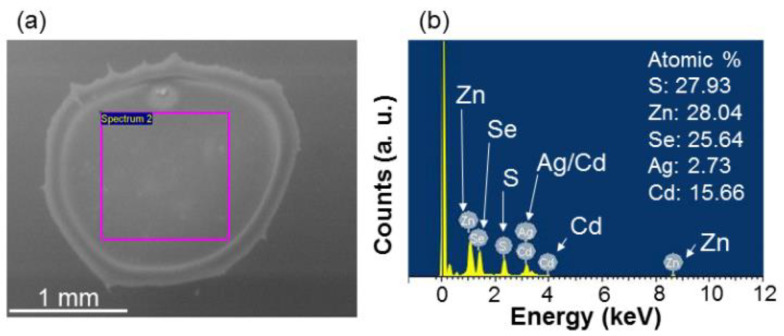
(**a**) SEM image of the dried drop-casted droplet on a GaN template with the same Ag NP and QD compositions as those of sample NP-GQD&RQD. The EDX scanning region is circled by the pink square. (**b**) EDX scanning intensities of various compositing elements of the Ag NPs and QDs. The atomic percentages of those recorded elements are shown in part (**b**).

As schematically illustrated in Figure 6(b1–b3), samples NP-GQD, NP-RQD, and NP-GQD&RQD are fabricated by drop-casting QD-linked Ag NPs (Ag nano-plates) onto PAH over-coated LEDs. In synthesizing Ag nano-plates, Ag seeds are first formed followed by a coating step to enlarge metal NP sizes and shape NP geometries [50]. The nano-plate diameter lies in the range of 25–60 nm and the thickness is ~10 nm. With a residual surfactant, i.e., citrate, the synthesized Ag nano-plates are negatively charged with the zeta potential at −35.5 mV. To make them become positively charged for attracting negatively charged QDs, they are capped with 5 k PEG to change the zeta potential into 43.2 mV. In fabricating sample NP-GQD (NP-RQD), we drop-cast 200 µL (50 µL) GQD (RQD) solution and 30 µL PEG-capped Ag nano-plate solution onto an LED for interaction and drying-up. For sample NP-GQD&RQD, we drop-cast 30 µL PEG-capped Ag nano-plate solution and the mixture of 200 µL GQD solution and 50 µL RQD solution onto an LED for interaction and drying-up. The QDs can be linked onto the PEG-capped Ag nano-plates before the solutions dry up. The particle concentration of the used Ag nano-plate solution is ~5.69 × 10^10^ mL^−1^. Because the LED epitaxial structure is opaque, to understand the localized surface plasmon (LSP) resonance behaviors of the Ag nano-plates in the samples, we fabricate the same surface structures as those of samples NP-GQD, NP-RQD, and NP-GQD&RQD on transparent GaN templates for measuring their transmission spectra (using a facility of JASCO Corporation with model V-670) to give the results shown in Figure 7. Here, all the transmission depression minima, i.e., the LSP resonance peaks, are close to 550 nm. The LSP resonance feature covers the emission wavelengths of the LED QW, GQD, and RQD, as indicated by the three vertical dashed lines. The insert of Figure 7 shows the scanning electron microscopy (SEM) image of sample NP-GQD&RQD, which is obtained by using a JEOL microscope (model JSM7001F). Here, we can clearly see Ag nano-plates and linked or un-linked QDs. However, we cannot differentiate GQD from RQD because their sizes are about the same. The LSP resonance of the Ag nano-plates can effectively couple with nearby GQD and/or RQD, but not the QWs because the top QW in the LED is located at a depth of >120 nm. To confirm that the samples are indeed overlaid with Ag NPs and QDs, energy-dispersive X-ray spectroscopy (EDX) scanning in SEM observation is performed by using a JEOL JSM-6510LV microscope and an Oxford Instruments Inca x-act EDX facility. However, in a direct EDX measurement on the surface of an LED sample, the obtained compositions can be dominated by those of the LED electrodes. Therefore, we drop-cast the NP solutions, which are used for fabricating sample NP-GQD&RQD, onto a GaN template for the EDX measurement. Figure 8a shows the SEM image of the dried NP droplet on the GaN template. Here, we can see parts of Ag NPs and QDs are accumulated at the border of the NP droplet. We choose the central area, as circled by the pink square in Figure 8a, for EDX scan. Figure 8b shows the EDX scanning results of the elements of S, Zn, Se, Ag, and Cd. The atomic percentages of those recorded elements are also shown in Figure 8b. The relatively lower atomic percentage of Ag can be attributed to the sediment of the heavier Ag NPs in the solution droplet such that the Ag signal of the planar EDX scan becomes weaker.

### 3.2. Device Characterization Results

**Figure 9:**
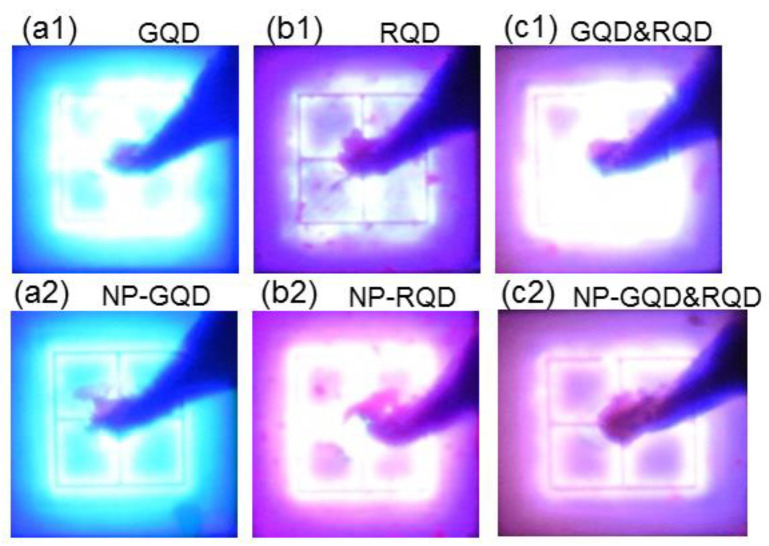
(**a1**)–(**c1**) and (**a2**)–(**c2**): Photographs of the lit LEDs of samples GQD, RQD, and GQD&RQD (NP-GQD, NP-RQD, and NP-GQD&RQD), respectively, when the injected current is 100 mA.

Figure 9(a1–c1) and Figure 9(a2–c2) shows the photographs of the lit LEDs of samples GQD, RQD, and GQD&RQD (NP-GQD, NP-RQD, and NP-GQD&RQD), respectively, when the injected current is 100 mA or the injected current density is 111.1 A/cm^2^. Here, the mixed light color of each sample can be seen. In the fabricated samples, the emissions of GQDs are relatively weaker because of the relatively lower emission efficiency of the purchased GQDs. This problem can be solved by reducing the surface state density on a GQD (as claimed by the QD manufacturer) or using a higher GQD concentration. In some photographs of Figure 9, we can clearly see the p-type contact geometry, which includes a square circuit, a cross, and a central circular area, all connected together. Moreover, the emission areas correspond to the square mesa regions of 300 µm × 300 µm in size.

**Figure 10:**
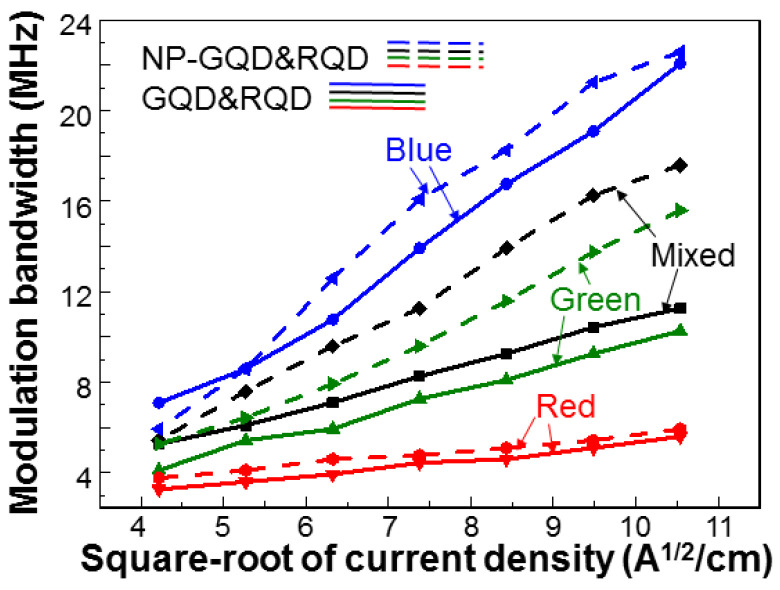
MBs of blue, green, red, and mixed lights in samples GQD&RQD (continuous curves) and NP-GQD&RQD (dashed curves) as functions of the square root of injected current density.

**Figure 11:**
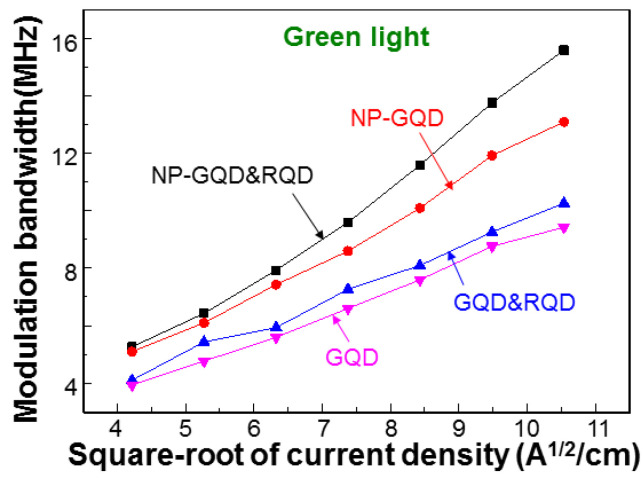
MBs of green light in all the samples with GQD as functions of the square root of injected current density.

**Figure 12:**
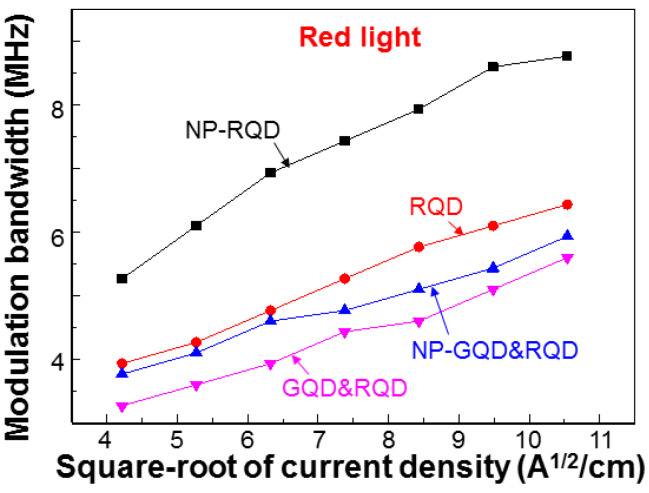
MBs of red light in all the samples with RQD as functions of the square root of injected current density.

Figure 10 shows the MBs of the blue, green, red, and mixed lights in samples GQD&RQD (continuous curves) and NP-GQD&RQD (dashed curves) as functions of the square-root of injected current density. The system used for measuring the MBs has been described in two previous publications of ours [41,42]. In either sample, the MB of green light is higher than that of red light. That of mixed light lies between blue and green lights. The smaller MB of red light, when compared with that of green light, is attributed to the lower carrier decay rate in RQD, which is caused by the stronger quantum confinement in RQD, when compared to GQD. Compared with the results of sample GQD&RQD, sample NP-GQD&RQD shows higher MBs for the converted and mixed lights, indicating the SP coupling effects of the Ag nano-plates. Figure 11 (Figure 12) shows the MBs of the green (red) light in all the samples containing GQD (RQD). In either Figure 11 or Figure 12, due to SP coupling, the MB is higher in a sample with Ag NP, when compared with a sample of the same QD structure but no Ag NP. Moreover, either with or without Ag NP, the MB of the green (red) light of a sample with GQD (RQD) only is always lower (higher) than that of a sample with both GQD and RQD. The MB variation trends of green and red lights are opposite. Such MB variations are caused by the energy transfer from GQD into RQD, as to be further discussed later. In Table 1, we show the MBs of blue, green, red, and mixed lights of all the samples under study when the injected current is 100 mA (~111.1 A/cm^2^ in injected current density). Here, the numbers inside the parentheses (curly brackets) for blue and green (blue and red) lights show the MB ratios with respect to sample GQD (RQD). The significantly larger MB ratios of green and red lights, when compared with those of blue light, confirm that the MB variations of green and red lights are caused by the SP coupling and/or energy transfer from GQD into RQD, instead of the MB variations of the fundamental blue light.

### 3.3. Discussions

**Figure 13:**
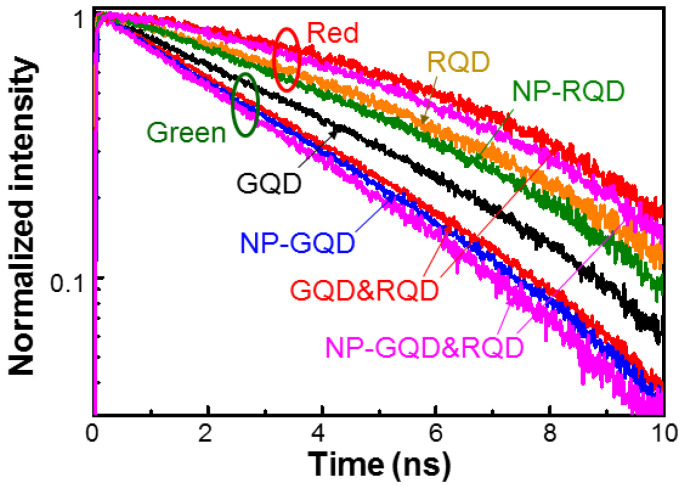
PL decay profiles of green and red lights in all samples under study.

When GQD and RQD are mixed for color conversion, both far- and near-field energy transfers from GQD into RQD occur. The far-field transfer means the RQD absorption of GQD emission. The near-field transfer is the FRET from GQD into RQD. The FRET can result in the faster decay of GQD carrier density and hence the MB increase in green light. On the other hand, both far- and near-field energy transfers from GQD into RQD can lead to the slower decay of RQD carrier density and hence the MB decrease in red light. The FRET can be confirmed by the measurement of the PL decay time of green light. Figure 13 shows the PL decay profiles of the green and red lights of the six samples under study. A PL decay measurement is excited by the second-harmonic (390 nm in wavelength) of a femtosecond Ti:sapphire laser (Coherent, Santa Barbara, USA, VERDI-8W). The time-resolved signal is monitored by a photon counter of 2.5 ps in temporal resolution (Acton Research Corporation, Acton, USA, SpectraPro 2150i). The PL decay rates of green light are generally higher than those of red light. Columns 2 and 3 of Table 2 show the calibrated PL decay times of green and red lights, respectively, in various samples. The PL decay times are calibrated based on an extended-exponential model [75]. The green PL decay time decreases from 4.095 ns in sample GQD to 3.453 ns in sample GQD&RQD through the FRET effect. The SP coupling in sample NP-GQD can effectively increase the carrier decay rate in GQD and hence reduce its PL decay time to 3.291 ns. Then, in sample NP-GQD&RQD, the combination of SP coupling and FRET leads to an even shorter green PL decay time at 3.095 ns. On the other hand, the far- and near-field energy transfers from GQD into RQD result in the increase in red-light PL decay time from 5.377 ns in sample RQD to 6.505 ns in sample GQD&RQD. With SP coupling in sample NP-RQD, the red-light PL decay time is reduced to 4.942 ns. Then, the combination of SP coupling and energy transfer brings us a larger red-light PL decay time at 6.052 ns. The FRET efficiency is usually defined as 1-τ_GQD&RQD_/τ_GQD_ [72]. Here, τ_GQD&RQD_ (τ_GQD_) represents the green-light PL decay time of a sample with GQD and RQD coexistent (the corresponding sample with GQD only). The FRET efficiencies of samples GQD&RQD and NP-GQD&RQD are shown in column 4 of Table 2. The FRET efficiency decreases from 15.7 to 6.0% when the SP coupling effect is introduced. SP coupling can reduce the FRET efficiency because the emission efficiency of the energy donor (GQD) is enhanced to compete with FRET for energy.

The almost linearly increasing trends of blue-light MB with the square root of injected current density, *J*, shown in Figure 10 indicate the consistency with the theoretical prediction of MB∝(*JB*/*qd*)^1/2^ shown in Equation (1). It is interesting to see that the MB variations of green and red lights with the square root of injected current density are also quasi-linear. The induced effects of SP coupling and/or energy transfer can change the values of B (the inverse of PL decay time) in QW, GQD, and RQD. As mentioned in Section 2.2, the inverse ratio of the square root of PL decay time in a QW with such a change under SP coupling is usually about the same as the MB ratio of the LED. However, for converted green and red lights, the inverse ratios of the square roots of PL decay times, as shown within the parentheses and curly brackets in Table 2, are quite different from the corresponding MB ratios, as shown within the parentheses and curly brackets in Table 1. These differences indicate that if other factors are fixed, the MB of a converted light is not linearly proportional to the PL decay rate of the acceptor. In other words, besides that of acceptor emission, the modulation responses of donor emission, acceptor absorption, and carrier relaxation play important roles in determining the MB of a converted light. This subject deserves further investigation. Nevertheless, a shorter PL decay time (a larger B) in the acceptor always leads to a higher MB for the converted light.

The MB of a mixed light depends on the MBs and the relative intensities of the light color components. In the current study, the blue light is stronger than either green or red light and hence the MB of the mixed light is dominated by that of the blue light. However, it is strongly influenced by the MBs of the green and red lights. With SP coupling, the MBs of the mixed lights are increased together with those of the green and red lights. With energy transfer from GQD into RQD, the MB of the mixed light in sample GQD&RQD or NP-GQD&RQD lies between those of the mixed lights in the corresponding pure GQD and pure RQD samples. The inclusion of red light always results in a smaller mixed-light MB. The LED MBs of hundreds of MHz have been reported when the mesa size is reduced to a few tens of microns for decreasing the RC time constant [41,42,43]. Since the modulation response of a converted light follows that of the fundamental LED emission, the MB of a converted light can also reach the level of hundreds of MHz in a micro-LED.

## 4. Conclusions

In summary, in this paper, we first discussed the fundamentals of the SP couplings with the QWs of an LED and its photon down-conversion QDs for enhancing the MBs of their emissions. The basic mechanisms for such an enhancement were explained in detail. The past results related to this subject were revisited for explaining those mechanisms, laying the foundation for the presentation of the new experimental data. Then, we demonstrated the MB enhancements of GQD- and RQD-based converted lights on blue-emitting LEDs through the SP coupling effects of linked synthesized Ag nano-plates. The surface of an Ag nano-plate was modified to become positively charged for linking with negatively charged GQD and RQD to guarantee their small separations and hence effective SP coupling. The energy transfer from GQD into RQD, including the mechanism of FRET, resulted in the MB increase (decrease) in green (red) light. The measurement results of the time-resolved PL of those emissions confirmed the behaviors of the MB enhancement. Based on the efforts of many researchers in the past several years, the MBs of LEDs and their down-converted lights have been significantly increased up to the GHz level, particularly in the configuration of a micro-LED. Although the technique of SP coupling has been used for enhancing the modulation responses of an LED and its down-converted light, more efforts are needed to fully utilize its advantages and further improve the modulation behaviors. In this regard, the application of QD-linked synthesized metal NPs overlaid on or embedded in an LED to induce strong SP coupling effects represents a new and effective technique for enhancing the MB of a down-converted light. Because of the large mesa size in the demonstrated LED devices, the obtained MBs in this paper are not as high as those in some of the cited publications. It is believed that when the presented SP coupling technique is applied to a micro-LED, its MB can become significantly higher, particularly when this SP coupling technique is used in addition to the methods proposed in those cited publications.

## Figures and Tables

**Table 1:** MBs of blue, green, red, and mixed lights in all the samples under study when the injected current is 100 mA. The numbers within parentheses and curly brackets show the ratios of MBs with respect to individual normalization base in each column.

Sample	Blue (MHz)	Green (MHz)	Red (MHz)	Mixed (MHz)
GQD	21.75 (1)	9.42 (1)	---	12.75
RQD	20.25 {1}	---	6.43 {1}	11.09
GQD&RQD	22.08 (1.02)	10.26 (1.09)	5.59 {0.87}	11.26
NP-GQD	22.74 (1.05)	13.09 (1.39)	---	18.75
NP-RQD	22.91 {1.09}	---	8.76 {1.36}	16.26
NP-GQD&RQD	22.58 (1.04)	15.58 (1.65)	5.93 {0.92}	17.58

**Table 2:** PL decay times of green and red lights, and the FRET efficiencies in the samples under study. The numbers within parentheses and curly brackets show the inverse ratios of the square roots of PL decay times with respect to individual normalization base in each column.

Sample	Green PL Decay Time (ns)	Red PL Decay Time (ns)	FRET Efficiency (%)
GQD	4.095 (1)	--	--
RQD	--	5.377 {1}	--
GQD&RQD	3.453 (1.09)	6.505 {0.91}	15.7
NP-GQD	3.291 (1.12)	--	--
NP-RQD	--	4.942 {1.04}	--
NP-GQD&RQD	3.095 (1.15)	6.052 {0.94}	6.0

## Data Availability

All the data supporting reported results can be found in the text of this paper.
